# Cortical Signal Analysis and Advances in Functional Near-Infrared Spectroscopy Signal: A Review

**DOI:** 10.3389/fnhum.2016.00261

**Published:** 2016-06-09

**Authors:** Muhammad A. Kamran, Malik M. Naeem Mannan, Myung Yung Jeong

**Affiliations:** Department of Cogno-Mechatronics Engineering, Pusan National UniversityBusan, South Korea

**Keywords:** hemodynamic response model, physiological noises, functional near-infrared spectroscopy, differential path length factor, resting-state functional connectivity

## Abstract

Functional near-infrared spectroscopy (fNIRS) is a non-invasive neuroimaging modality that measures the concentration changes of oxy-hemoglobin (HbO) and de-oxy hemoglobin (HbR) at the same time. It is an emerging cortical imaging modality with a good temporal resolution that is acceptable for brain-computer interface applications. Researchers have developed several methods in last two decades to extract the neuronal activation related waveform from the observed fNIRS time series. But still there is no standard method for analysis of fNIRS data. This article presents a brief review of existing methodologies to model and analyze the activation signal. The purpose of this review article is to give a general overview of variety of existing methodologies to extract useful information from measured fNIRS data including pre-processing steps, effects of differential path length factor (DPF), variations and attributes of hemodynamic response function (HRF), extraction of evoked response, removal of physiological noises, instrumentation, and environmental noises and resting/activation state functional connectivity. Finally, the challenges in the analysis of fNIRS signal are summarized.

## Introduction

Near-infrared spectroscopy (NIRS) is an emerging non-invasive brain-imaging methodology that utilizes near-infrared (NIR) light of 650–900 nm to determine cerebral oxygenation, blood flow, and the metabolic status of a localized region of the brain (Saager and Berger, 2008; Yamada et al., 2009; Khan et al., 2014; Molavi et al., 2014; Santosa et al., 2014; Naseer and Hong, 2015). Activation in a particular part of the brain causes an increase in the regional cerebral blood flow (rCBF) (Zhang et al., 2011b; Umeyama and Yamada, 2013; Kopton and Kenning, 2014). The rate of rCBF increase exceeds that of the regional cerebral oxygen metabolic rate (rCMRO2), which is the major cause of de-oxy hemoglobin (HbR) decrease in venous blood (Sitaram et al., 2009). Thus, cortical activation causes an increase in total hemoglobin (HbT) and oxy-hemoglobin (HbO), with a corresponding decrease in HbR. The absorption of NIR light changes with changes in the concentration of HbO and HbR during activation and rest periods. The attenuation of NIR light due to the absorption change reflects, according to the modified-Beer Lambert law (MBLL), the concentrations of HbO and HbR. Among neuro-imaging modalities, functional near-infrared spectroscopy (fNIRS)'s simplicity, portability, low cost, good temporal resolution (suitable for real-time imaging), and high signal-to-noise ratio make it a favorable option (Hu et al., 2013; Chang et al., 2014; Herff et al., 2014). fNIRS also has been considered as a potential multi-modality imaging methodology (Yunjie and Blaise, 2012). One disadvantage of fNIRS, however, is its low penetration depth. Details on the pros and cones of fNIRS can be found in Gervain et al. (2011), Barati et al. (2013), and Tak and Ye (2013).

The increase in HbR at particular area of brain is an indicator of the neuronal activity in nearby area. The detection of the neuronal activation in a particular cortical area is nothing but extraction of a specific waveform in the hemodynamic response (HR) (Ciftçi et al., 2008). In past, the canonical hemodynamic response function (cHRF) is frequently be used as desired impulse response in hemodynamic signal. Of course, it could vary in its shape, time to peak, relaxation time, and full width half maximum (FWHM). These variation in the characteristics of HRF are observed in different brain areas, among subjects and on repetition of trials. Such variations in the attributes of cHRF measured by fNIRS, has been observed in HbO concentration changes (Hong and Nugyen, 2014). The major cause of this phenomenon is the brain's continuous consciously/unconsciously processing for several tasks at the same instant of time. Even if the subject is instructed to relax and sit comfortably during an experiment, the brain consciously or unconsciously processes for many past, present and future events. Several studies have reported such findings during the analysis of fNIRS data. The difference in the dynamical shape of HRF during event-related motor and visual paradigms revealed that the peak times of HbO, HbR, and total hemoglobin (HbT) for visual paradigm are approximately equal unlike for motor paradigm (Jasdzewski et al., 2003). An additional source of such variations in hemodynamic signal measured through fNIRS, could also be as a result of certain artifacts (Yamada et al., 2009; Umeyama and Yamada, 2013). The artifacts could be related to instrumentation noise, not proper fixation of NIRS optodes, and motion of subjects likewise body tilt, breathing hold, and head nodding etc. (Yamada et al., 2009; Robertson et al., 2010; Umeyama and Yamada, 2013). Among these considerations, there is another factor that can affect the shape of HRF. This factor is known as differential path length factor (DPF). It determines the additional distance traveled by light photon due to scattered behavior of brain tissues. It is found that the wavelength dependent DPF and age can also affect the characteristics of HR (Duncan et al., 1996). A mismatch between these features could result as a decrease in the detection performance (Ciftçi et al., 2008).

**Figure 1:**
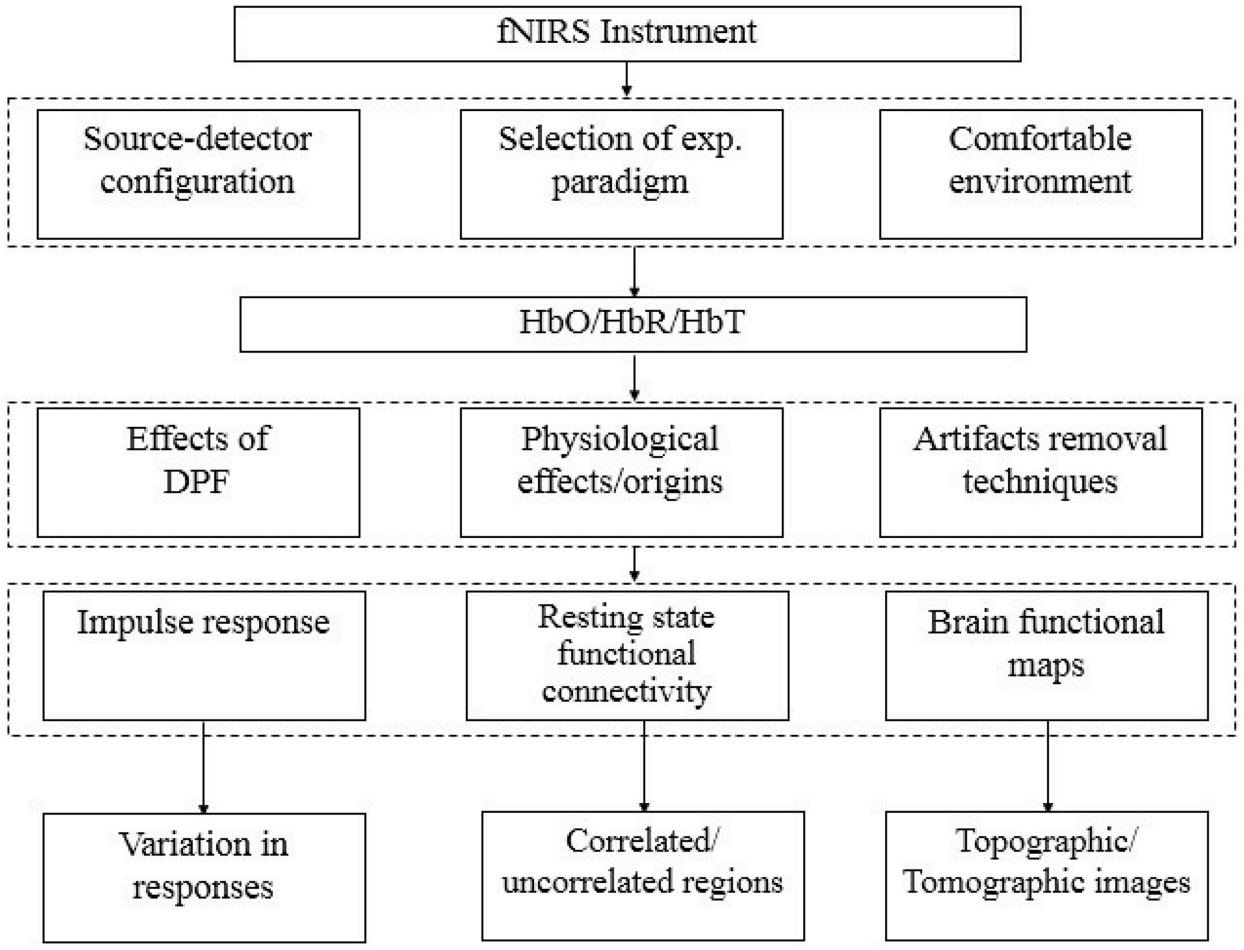
**Stages of fNIRS signal analysis**.

Additionally, NIRS signals include physiological noises associated with heart beats, respiration rhythms and low-frequency fluctuations. A special algorithm that not only to suppress physiological signals present in optical signals, measured through fNIRS, but also other unwanted (activation not related to experimental paradigm) signals due to continuous brain processing, therefore is required. In fNIRS signal analysis, most of studies have been reported in relation with reduction of physiological and instrumentation noises or to extract neuronal activation related waveform. But recent research in this field has also been turned toward the analysis of functional connectivity of brain regions during resting states (Lu et al., 2010; Hu et al., 2013). Additionally, does this resting state connection stays during task periods of a particular region (Zhang et al., 2011b; Hu et al., 2012, 2013)? Until now fNIRS has a limitation that NIRS optodes cannot cover full skull at once to study/analyze complete functional connectivity (Lu et al., 2010). Figure 1 summarizes different subfields in the area of fNIRS signal analysis for development of a standard methodology.

## Brain optical signal

### Data acquisition and pre-processing

**Figure 2:**
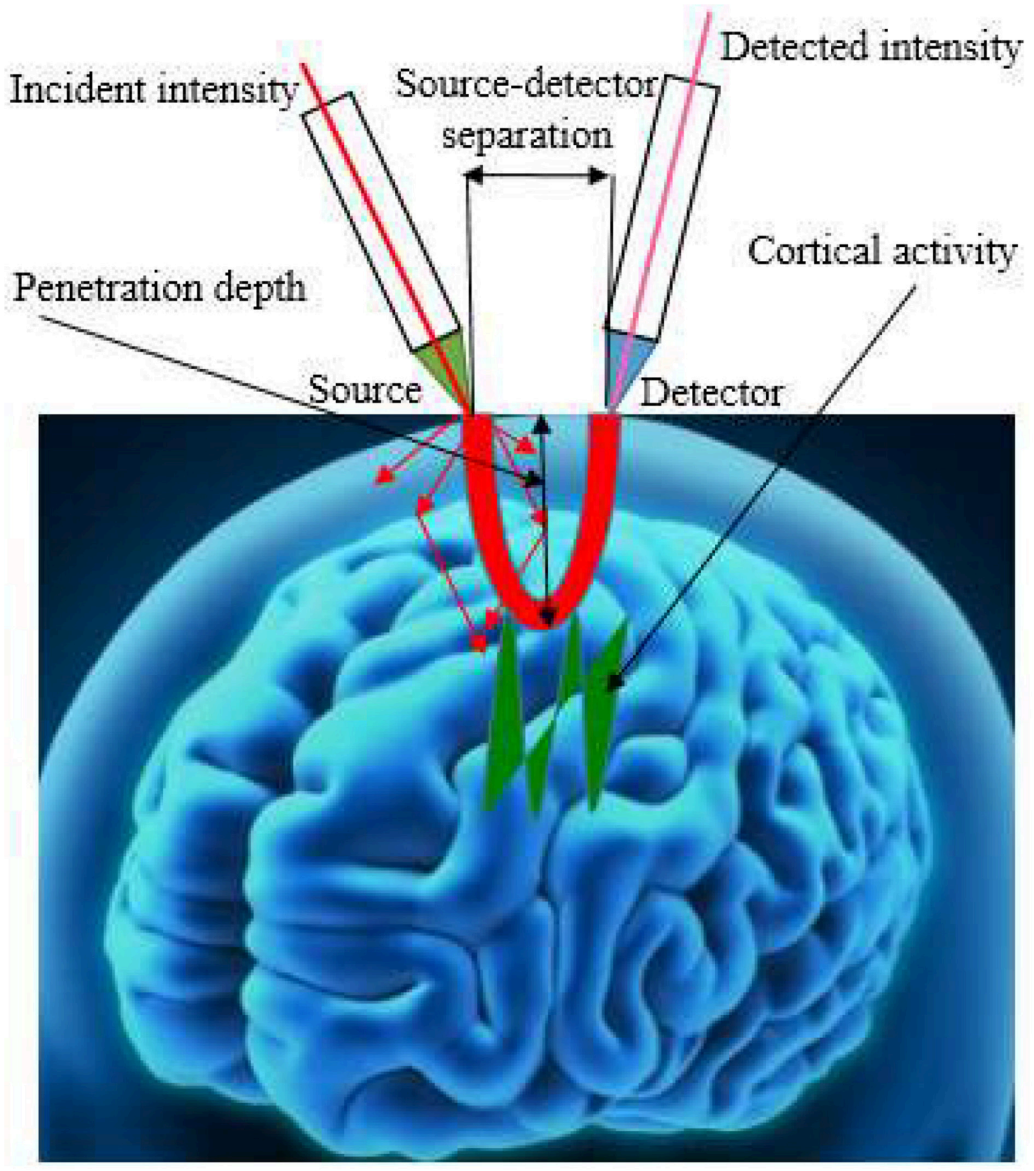
**The geometry of fNIRS signal acquisition**.

In recent years, different types of NIRS imaging systems have been developed, which can be grouped into continuous wave (CW), frequency domain (FD), and time-resolved (TR) categories. CW-fNIRS instrument measures the concentration changes of HbO, HbR, and total hemoglobin with assumption that scattering remains constant, while FD-fNIRS and TD-fNIRS detects the absolute concentrations of HbO and HbR. TD-fNIRS is based on the principle of time of flight measurement and the most expensive instrument. The central differentiating element among these instruments is the estimation of the path length traveled by the photon due to scattering. The CW system, the least expensive, provides relative-change information in the forms of the concentrations of HbO and HbR, and was the version utilized most frequently. Figure 2 shows the geometry of fNIRS signal acquisition.

The transportation of light photon through tissue is a complex process. When photon of different wavelengths incident on tissues, the characteristics of the detected light depends upon the combination of scattering, absorption, and reflection (Cope and Delpy, 1988). It can be modeled using the modified Beer-Lambert law given as

(1) $$\begin{array}{l} I_{o}\left(\lambda \right)=I_{in}\left(\lambda \right)e^{-\mu_{a}\left(\lambda \right)dDPF\left(\lambda \right)+G\left(\lambda \right)} \end{array}$$

where *I*_*in*_(λ), *I*_*o*_(λ) are the incident and the detected light respectively, μ_*a*_(λ) is the absorption, *d* is the source detector separation, *DPF*(λ) is the DPF, and *G*(λ) is the geometry-dependent parameter. The first step to express chromophore changes from optical signal is to find the optical density (OD) defined as (Cope and Delpy, 1988; Duncan et al., 1996; Kamran and Hong, 2014)

(2) $$\begin{array}{l} OD=\ln\;\left(\frac{I_{in}\left(\lambda \right)}{I_{o}\left(\lambda \right)}\right)=\mu_{a}\left(\lambda \right)dDPF\left(\lambda \right)+G\left(\lambda \right) \end{array}$$

or

(3) $$\begin{array}{l} \Delta OD=\Delta \mu_{a}\left(\lambda \right)dDPF\left(\lambda \right)+G\left(\lambda \right) \end{array}$$

Considering two chromophores, i.e., HbO and HbR and assuming the phenomenon of scattering to be constant,

(4) $$\begin{array}{l} \Delta OD^{\lambda_{i}}=\left(\varepsilon_{HbO}^{\lambda_{i}}\Delta HbO+\varepsilon_{HbR}^{\lambda_{i}}\Delta HbR\right)dDPF\left(\lambda_{i}\right)+G \end{array}$$

where λ_*i*_ is the wavelength of the incident light and $\varepsilon_{HbO}^{\lambda_{i}}$ and $\varepsilon_{HbR}^{\lambda_{i}}$ are the extinction coefficients of HbO and HbR, respectively. By considering two different wavelength of light, the above equation could be rearranged as follows (Ye et al., 2009; Kamran and Hong, 2013; Santosa et al., 2013)

(5) $$\begin{array}{l} \Delta HbO^{i}\left(k\right)=\frac{\left(\varepsilon_{HbO}^{\lambda_{1}}\frac{\Delta OD^{\lambda_{2}}\left(k\right)}{DPF^{\lambda_{2}}}\right)-\left(\varepsilon_{HbR}^{\lambda 2}\frac{\Delta OD^{\lambda_{1}}\left(k\right)}{DPF^{\lambda_{1}}}\right)}{d^{i}\left(\varepsilon_{HbR}^{\lambda_{1}}\varepsilon_{HbO}^{\lambda_{2}}-\varepsilon_{HbR}^{\lambda_{2}}\varepsilon_{HbO}^{\lambda_{1}}\right)}, \end{array}$$

(6) $$\begin{array}{l} \Delta HbR^{i}\left(k\right)=\frac{\left(\varepsilon_{HbO}^{\lambda_{2}}\frac{\Delta OD^{\lambda_{1}}\left(k\right)}{DPF^{\lambda_{1}}}\right)-\left(\varepsilon_{HbO}^{\lambda_{1}}\frac{\Delta OD^{\lambda_{2}}\left(k\right)}{DPF^{\lambda_{2}}}\right)}{d^{i}\left(\varepsilon_{HbR}^{\lambda_{1}}\varepsilon_{HbO}^{\lambda_{2}}-\varepsilon_{HbR}^{\lambda_{2}}\varepsilon_{HbO}^{\lambda_{1}}\right)}, \end{array}$$

where Δ*HbR*^*i*^(*k*) and Δ*HbR*^*i*^(*k*) are relative concentration changes of HbO and HbR respectively, *k* is the discrete time, *i* represents the *i*th-channel of emitter-detector pair, λ_1_ and λ_2_ represent two different wavelengths, $\varepsilon_{HbO}^{\lambda_{1}}$, $\varepsilon_{HbR}^{\lambda_{1}}$, $\varepsilon_{HbO}^{\lambda_{2}}$, and $\varepsilon_{HbR}^{\lambda_{2}}$ indicates the extinction coefficients of HbO and HbR at two different wavelengths respectively, $\Delta OD^{\lambda_{j}}\left(k\right)$ is the optical density variation at *k*th-sample time at particular wavelength (*j* = 1, 2), *d*^*i*^ is the source-detector separation and $DPF^{\lambda_{j}}$ is the DPF at particular wavelength (*j* = 1, 2).

## Hemodynamic response analysis

### Effects of differential path length factor

The scattering behavior of human brain tissue to NIR light entails that DPF is required to correct the reading observed through fNIRS. Initially it was in practice to use DPF value between 3 and 6. Later, time of flight methodology or intensity modulated spectroscopy were used to estimate the values of DPF. Duncan et al. (1995) found the DPF values for adult heads for four different wavelengths in a population of 100 (50 males and 50 females) subjects. It was found that the value of DPF are 6.51 ± 1.13, 6.53 ± 0.99, 6.26 ± 0.88, and 5.86±0.98 for 690, 744, 807, and 832 nm, respectively. The magnitude of the DPF determines the magnitude of the calculated concentration changes (Kohl et al., 1998). Therefore, DPF has an important role for any instrument claiming accurate measurement of chromophores changes. Kohl et al. (1998) has used the key idea that DPF is proportional to the rate of change of absorbance with respect to absorption. Thus, they found wavelength dependent DPF by the ratio that rate of change of absorbance to the absorption spectrum of arterial blood. Duncan et al. (1996) analyzed 283 subjects (age between 1 day and 50 years) and developed equations to express DPF values as a function of age at different wavelengths. Their results summarizes the DPF values for four different wavelengths as under;

(7) $$\begin{array}{l} DPF^{690}\;=\;5.38\;+\;0.049^{*}\left(A^{0.877}\right), \end{array}$$

(8) $$\begin{array}{l} DPF^{744}\;=\;5.11\;+\;0.106^{*}\left(A^{0.723}\right), \end{array}$$

(9) $$\begin{array}{l} DPF^{807}=4.99+0.067^{*}\left(A^{0.814}\right), \end{array}$$

(10) $$\begin{array}{l} DPF^{832}=4.67+0.062^{*}\left(A^{0.819}\right). \end{array}$$

But CW-NIRS systems could be used for different wavelengths for their equipment, thus a general equation was the requirement that could be used for any wavelength and for any age. Schroeter et al. (2003) analyzed fNIRS data from 14 young (23.9 ± 3.1 years old) and 14 elderly (65.1 ± 3.1) subjects and suggested that DPF is not only effected by age but also with different brain regions. They concluded that the hemodynamic response can be decreased by age in the frontal association cortex during functional activation and proposed to calculate effect size to analyze age-related effects in fNIRS studies. Scholkmann and Wolf (2013) realized this need and fitted a polynomial of degree three on the available data set for the values of DPF. They represented DPF as a function of wavelength and age as under

(11) $$\begin{array}{l} DPF\left(\lambda ,\;A\right)=\alpha \;+\;\beta A^{\Gamma }\;+\;\delta \lambda^{3}+\;\varepsilon \lambda^{2}\;+\;\varsigma \lambda . \end{array}$$

The values of the unknown parameters were found by using Levenberg-Marquardt algorithm (LMA) and least absolute residuals (LAR). There results suggested that

(12) $$\begin{array}{rcl} DPF\left(\lambda ,A\right) & = & 223.3+0.05624\;A^{0.8493}-5.723^{*}10^{-7}\lambda^{3}\\ +\;0.001245\lambda^{2}-0.9025\lambda . \end{array}$$

The above equation is a generalized form of DPF correction depending upon age and wavelength. This equation is advantageous to use for any researcher because any one can easily evaluate the DPF value at any wavelength and age. The published articles presenting the values of DPF for different age and wavelengths have been summarized in Table 1.

**Table 1:** **The published references for DPF values**.

**References**	**Cortex**	**Subjects**	**Age**	**Wavelengths**	**S-D separation**
van der Zee et al., 1992	Temporal frontal	10 adults, 10 Infants	22~54 Y	761 nm	2.5 cm
Duncan et al., 1995	Left forehead	100 adults, 35 Infants	21~ 9 Y and Infants	690, 744, 807, 832 nm	>4 cm
Duncan et al., 1996	Left forehead	283	1 day ~50 Y	690, 744, 807, 832 nm	4.5 cm
Cooper et al., 1996	Temporal frontal	19	23~38 weeks	730 and 830 nm	4.9 cm
Kohl et al., 1998	Right occipital	10	23~40 Y	700–1000 nm	3 cm
Scholkmann and Wolf, 2013	NA	NA	Applicable to all	Applicable to any	NA

### Variations in HRF pattern

The neural activation indication, measured through fNIRS, may be confounded with individual anatomical or systemic physiological sources of variance (Heinzel et al., 2013). Generally, inter-subject variability is due to the individual's differences in anatomical factors likewise skull and cerebrospinal fluid (CSF) structure, vessels distributions, and the ratios of the arteries and veins. Barati et al. (2013) observed that the variability in the stimulus condition for HbO was revealed in the slope, amplitude, and timing of the peak response. Jasdzewski et al. (2003) analyzed the difference in the dynamical shape of HRF during event-related motor and visual paradigms. Their results revealed that the peak times of HbO, HbR, and total hemoglobin (HbT) for visual paradigm are approximately equal unlike for motor paradigm (Jasdzewski et al., 2003). Additionally, their results have been analyzed for different values of source-detector separation. But if the source-detector separation is greater than 3 cm than the results are not much affected by DPF values (Duncan et al., 1996). Power et al. (2012) analyzed two very important questions: (1) is it possible to distinguish activation task from baseline or from other tasks? And if so (2) are the spatiotemporal characteristics of the response consistent across sessions? Their results concluded that the mental arithmetic tasks can be distinguished from base line but the characteristics of the response changes from session to session. Hong and Nugyen (2014) analyzed 19 subjects to conclude variations in the impulse responses at three different brain regions, somatosensory cortex (SC), motor cortex (MC), and visual cortex (VC). Their findings suggest that the activation- and the undershoot-peak of the HbO in MC are higher than those in SC and VC. Additionally, the time-to-peaks of the HbO in three brain regions are almost the same (about 6.76 76 ± 0.2 s) and the time to undershoot peak in VC is the largest among three.

### Constrained basis set

The detection of cortical activation related waveform from neuroimaging discrete signal is nothing but a search for a consistent and specific wave pattern (Koray et al., 2008). This is equaling to fit the measured signal to a known waveform up to certain accuracy. A mismatch of such fitting might lead to misleading results. The cHRF attributes includes magnitude of initial dip, time to the first peak, time to the undershoot, magnitude of the undershoot etc. In literature, cHRF consisting of two gamma functions have been used most frequently. In which, first peak is to tackle the main response and second peak is for undershoot after the response. Likewise fNIRS, functional magnetic resonance imaging (fMRI) modeling requires flexible HRF modeling, with the HRF being allowed to vary spatially, on repetition of trials and between subjects (Woolrich et al., 2004). Thus, a Bayesian constrained frame work is described in Woolrich et al. (2004) to best choose the HRF in the measured data. Koray et al. (2008) proposed constraint GLM model parameters such as main response (time to first peak) must be within 3–8 s, not more than one positive peak, not more than two dips, initial dip magnitude must be lesser than quarter of the magnitude of onset, an undershoot after 2–8 s of time to peak and magnitude of post stimulus undershoot must be lesser than half of the magnitude of onset. A 3D volume of parameter values of the canonical basis set then supposed as prior distribution in the Bayesian analysis. Finally, Gibbs sampling in this volume is used to find out the parameter of interest. This method is advantageous as it tries to constraint the basis set with possible reduction in solution space. The solution space is reduced on the basis of HRF physical properties reported in fMRI data in past.

### Extraction of evoked-response

Jobsis (1977) was the first to present an idea that there is a possibility to detect changes of cortical oxygen using NIR light. Later, Cope and Delpy (1988) designed an NIR system with four different wavelengths (778, 813, 867, and 904 nm). It is well-known fact that the neuronal activity generates an early de-oxygenation in a particular area of the brain from where the activity is started. The characteristics of HRF include an early rise after 1–2 s of stimulation and reaches to peak around 5–6 s. Finally, it starts to drop down and reaches a base line level after having a slight undershoot. The total duration of HRF for impulse stimulation is around 26–30 s. Friston et al. (1994) introduced the statistical parameter mapping (SPM) software for fMRI signal analysis, modeling the oxygen dependent signal as linear combination of two Gamma functions.

**Figure 3:**
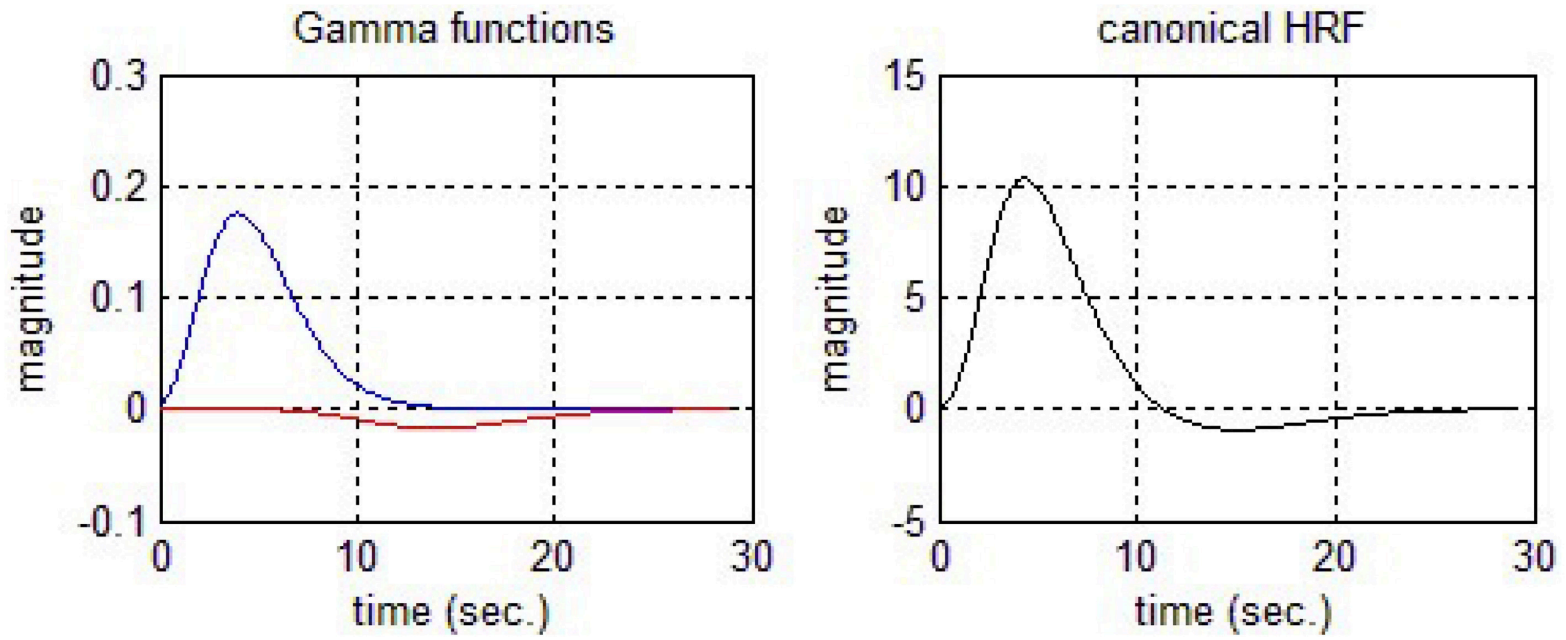
**Canonical hemodynamic response function (cHRF)**.

This two Gamma function model is most frequently used to account the first peak and final undershoot of the oxygen dependent waveform. Figure 3 displays two Gamma functions (on left side of Figure 3) and final cHRF by employing their linear combinations (right side of Figure 3). The standard values to generate the shape of cHRF are given in SPM (Friston et al., 1994, 1998). The mathematical form to generate this type of HRF is described below

(13) $$\begin{array}{l} HRF\left(k\right)=h{\left(k\right)}^{*}u\left(k\right), \end{array}$$

(14) $$\begin{array}{l} h\left(k\right)=\left[\frac{k^{\alpha_{1}-1}\beta_{{}_{1}}^{\alpha_{1}}e^{-\beta_{1}k}}{\Gamma \left(\alpha_{1}\right)}-\frac{k^{\alpha_{2}-1}\beta_{{}_{2}}^{\alpha_{2}}e^{-\beta_{2}k}}{6\Gamma \left(\alpha_{2}\right)}\right] \end{array}$$

where *u* is the experimental paradigm, h represents the cHRF, α_1_ is the delay of response, α_2_ is the delay of the undershoot, β_1_ is the dispersion of the response, β_2_ is the dispersion of the undershoot and Γ represents the Gamma distribution. Boynton et al. (1996) presented the idea to model neuronal related fNIRS waveform by employing only one Gamma function with two free parameters.

In contrast with fMRI, fNIRS observed signal is contaminated with several physiological noises. In most of studies, such signals are filtered out in pre-processing steps with known cutoff frequencies. But, Prince et al. (2003) modeled such signals as a linear combination of different sinusoids. In past several studies have modeled HRF using the model presented in Equations (13) and (14) in GLM based analysis framework. Jasdzewski et al. (2003) analyzed impulse response attributes in the form of a linear model. Later, Koh et al. (2007) introduced a functional optical signal analysis (fOSA) software based upon the GLM methodology. Plichta et al. (2006, 2007) presented the functional brain maps of visual cortex by employing GLM methodology with ordinary least square estimation (OLSE) to extract the values of activity strength parameters. Taga et al. (2007) analyzed the effects of source-detector separation to extract neuronal activity related fNIRS signal. Koray et al. (2008) estimated HRF by fitting constrained parameters of cHRF in Bayesian framework. NIRS-SPM (Ye et al., 2009) is the extension of SPM (Friston et al., 1994, 1998), frequently been used in fMRI analysis. This software package has employed the concept of GLM with known regressors to decompose the measured NIRS time series. The brain signal model could be represented mathematically in the form of known n-regressors,

(15) $$\begin{array}{l} y^{i}\left(k\right)=x_{1}\left(k\right)\beta_{1}+x_{1}\left(k\right)\beta_{2}+\cdots +x_{n}\left(k\right)\beta_{n}. \end{array}$$

The GLM methodology has been employed quite frequently while analyzing the fMRI time series. For this purpose, a basis set including predicted HRF (pHRF) and a base line correction is been used. Furthermore, the temporal and dispersion derivatives have been added to tackle the temporal and spatial effects in HRF (Friston et al., 1994, 1998). Likewise fMRI, fNIRS instrument monitors the concentration changes of HbO/HbR, thus an identical regression vector with fMRI is used in optical signal analysis. But fNIRS time series has additional challenges of existence of physiological signals in the measured waveform. Thus, Abdelnour and Huppert (2009) described the basis set as linear combination of pHRF, a base line correction and three sinusoids for physiological signals. Hu et al. (2010), supposed regression vector to be a combination of five components; pHRF, a baseline correction and remaining three forms a set of high pass filter with cut-off frequency 0.0006 Hz. Zhang et al. (2011b, 2012) introduced the use of recursive algorithms for better extraction of neuronal related concentration changes in observed fNIRS data. Aqil et al. (2012a) supposed fNIRS signal in a standard GLM framework with estimation of activity strength parameters using recursive algorithm. They modeled the fNIRS time series as a linear combination of pHRF, first derivative of pHRF (temporal derivative), second order derivative of pHRF (dispersion derivative) and a base line correction. Kamran and Hong (2013) has modified the method and analyzed the measured optical data in the form of linear parameter varying approach with a recursive technique that can estimate activity strength parameters in a Lagrangian framework. Scarpa et al. (2013) presented the idea to consider a reference channel with a source-detector separation of <0.7 cm. This reference channel contains only the physiological signals and useful component from nearby channel could be extracted by subtracting the data measured through reference channel. Later, Kamran and Hong (2014) modeled cortical signal in the form of auto-regressive moving average with exogenous signal (ARMAX). The physiological signal have been considered as a known amplitude and frequencies and variation in HRF is modeled by employing ARMA model. Hong and Nugyen (2014) has used the same basis set as described in Aqil et al. (2012b) to model impulse response as a state-space model.

Independent component analysis (ICA) is a powerful blind signal processing technique (Katura et al., 2008). It can extract independent components from measured discrete series by using statistical concepts. Morren et al. (2004) analyzed and detected fast neuronal signal with a source-detector separation of 3 cm using ICA technique. Zhang et al. (2013) has employed ICA methodology to explore the existence of particular wave form, modeled as Gamma variants. Similarly, Santosa et al. (2013) has used ICA to extract the pHRF from regression vector including pHRF, a baseline correction and physiological noises. NIRS data analysis is performed in medical field as well for detection of different brain diseases. Machado et al. (2011) have used the GLM methodology to estimate the existence of hemodynamic responses to epileptic activity. As a general comparison, the most of above methods could be analyzed on the basis of computational cost. For example, ICA needs more computational cost as compared to ordinary/recursive least squares estimation algorithm for unknown parameter estimation. Initial dip is an important and most crucial attribute and indicator of neuronal activity. It gives the idea of a particular location which is involved in originating the said activity. Thus, using Gamma functions as basis set could be more advantageous if the factor of initial dip is added and analyzed. Table 2 summarizes the list of studies describing methodologies to extract the neuronal activity related wave pattern from fNIRS signal.

**Table 2:** **Signal processing methodologies for extraction of evoked-hemodynamic response**.

**References**	**Methodological details**
Jobsis, 1977	Possibility to detect changes of cortical oxygen using NIR light.
Cope and Delpy, 1988	Design of NIR system with four wavelengths (778, 813, 867, and 904 nm) with applying modified Beer-Lambert law for data conversion.
Friston et al., 1994	Statistical parameter mapping software for fMRI but later used for fNIRS data analysis with modifications.
Boynton et al., 1996	HRF model with one Gamma function with two free parameters.
Prince et al., 2003	Biological signals modeled as sum of sinusoids.
Jasdzewski et al., 2003	Impulse response, initial dip, and time to peak analysis in fNIRS signal.
Koh et al., 2007	A software functional optical signal analysis (FOSA) was introduced based on GLM methodology.
Plichta et al., 2006, 2007	GLM methodology with ordinary least square estimation to generate functional maps of visual cortex.
Taga et al., 2007	Analysis of effect of source-detector separation to fNIRS hemodynamic response.
Koray et al., 2008	Estimation of constrained HRF parameters in Bayesian frame work.
Abdelnour and Huppert, 2009	GLM based methodology with Kalman filter to estimate handedness.
Ye et al., 2009	GLM based NIRS-SPM software package for analysis of fNIRS data.
Hu et al., 2010	Brain functional maps by using GLM and Kalman filtering.
Zhang et al., 2011b	Recursive least squares (RLS)-empirical mode decomposition for noise reduction.
Zhang et al., 2012	RLS estimation with forgetting factor to remove physiological noise.
Aqil et al., 2012a	GLM and RLSE for estimation of brain functional maps.
Aqil et al., 2012b	Generation of cHRF using state-space approach.
Scarpa et al., 2013	Reference channel based methodology for estimation of evoked-response
Santosa et al., 2013	ICA methodology to estimate pre-defined cortical activation signal.
Kamran and Hong, 2014	Linear parameter varying model and adaptive filtering to estimate HRF and functional maps of brain.
Barati et al., 2013	Principle component analysis to continuous fNIRS data (using spline method).
Kamran and Hong, 2014	Auto-regressive moving average model with exogenous signal (ARMAX) model for cortical activation estimation.
Hong and Nugyen, 2014	State-space model for impulse response using fNIRS.

### HRF model using state-space model

fNIRS measured time series is a discrete data series that could be converted into state-space model for further analysis. Aqil et al. (2012b) summarizes the state space model of optical time series using standard subspace-based approach, but the numeric values of final matrices were not been displayed. The brain model used in their work is similar with Aqil et al. (2012a). Later, Hong and Nugyen (2014) converted fNIRS cortical signal model (same as Aqil et al., 2012a) into state-space model by using standard subspace based approach. They have summarized the mathematical derivation as well and finally they used built in Matlab function to extract final state-space model and matrices of order six. They have also summarized the numeric values of finalized matrices for different brain regions as well. Kamran and Hong (2013) presented the idea that linear parameter varying model could be beneficial to tackle the time varying characteristics of the human brain signal. In their work, the measured optical data is modeled as a state-space model whose matrices are dependent upon time varying parameter. But a final state-space model has not been reported in their work. Modeling using state space methods could be beneficial over other estimation methodologies with recursive algorithms because the analysis in state space model would be much easier if a model is developed that can cater the attributes of HRF as variable parameters.

### Physiological noises

fNIRS data analysis incorporates an additional challenge of temporal correlation presented in the data due to the physiological signals. The physiological noises includes cardiac beat, respiration rhythm and low frequency fluctuations known as Mayer waves. In most of studies, the physiological signals are pre-filtered by using standard signal filtering techniques. Prince et al. (2003) has presented the idea to model the biological signals as a set of sinusoids. Zhang et al. (2007) has proposed to nullify the effects of global interferences by using multi-separation probe configuration (placing a detector close to the source) and adaptive filtering. Abdelnour and Huppert (2009) included physiological signals as known regressors in their regression set. Hu et al. (2010) have added a set of high pass filter with cut-off frequency 0.0006 Hz to tackle the physiological signals. Zhang et al. (2011b) analyzed multi-distant source-detector separation further by decomposing short distance source-detector measurement into intrinsic mode functions (IMFs). An estimate of global interference is derived by analyzing weight coefficients of IMFs. Cooper et al. (2012) has analyzed the simultaneous fNIRS and fMRI recordings to reduce the physiological effects. They calculated the variance of the residual error in a GLM of the base line fMRI signal and the observed variance is reduced by incorporating NIRS signal in the model. Zhang et al. (2012) presented to remove physiological effects from simulated fNIRS data set of near and far detectors using recursive algorithm. Yamada et al. (2012) proposed that functional and systemic responses could be separated on the basis of a negative and positive linear relationship between HbO and HbR changes of the functional and the systemic signals. Later, Kirilina et al. (2012) included an additional predictor to account for systemic changes in the skin to analyze time course, localization and physiological origin of task related superficial signals in fNIRS measured data. They found that skin blood volume depends upon the cortical state. Additionally they found that origin of the task related systemic signals in fNIRS are co-localized with veins draining the scalp. Frederick et al. (2012) generated regressors for systemic blood flow and oxygenation fluctuation effects by applying a voxel-specific time delay to concurrently acquired fNIRS-fMRI time series. Kamran and Hong (2014) added three sinusoids with known frequencies and amplitudes as exogenous signal in their ARMAX frame work. It is very important to find out the frequencies and amplitudes of existing sinusoid in measured data in addition with its origin. Since, the frequency of experimental paradigm coincident with physiological noises could result in the generation of harmonics. Most of the previous studies were using a generic idea regarding a fixed frequency and amplitude of known sinusoid to cater the physiological signals in fNIRS data. A recently published Kamran et al. (2015) is more advantageous estimation algorithm because it allows the user to estimate the frequency and amplitude of physiological signals automatically from measured data instead of using a fix pattern. The studies related to removal of physiological noises from fNIRS signal have been summarized in Table 3 describing cortical area, number of subjects, nature of mental task, methodology used and source-detector separation.

**Table 3:** **Physiological noise estimation and reduction in fNIRS measured signal**.

**References**	**Cortex**	**Subjects**	**Task**	**Methodology**	**S-D separation**
Prince et al., 2003	motor	5	Hand tapping/Rest	Stochastic model with extended Kalman filter	>2 cm
Zhang et al., 2007	Five layer slab model	Simulated data	Block design paradigm	Multi-separation probe configuration and Monte Carlo	1.5~4.5 cm
Abdelnour and Huppert, 2009	Motor	3	Finger tapping	GLM with Kalman estimator	3.1 cm
Zhang et al., 2011b	Five layer slab model	Simulated data	Block design paradigm	Multi-distance approach with empirical mode decomposition	0.1 cm
Zhang et al., 2012	Five layer slab model	Simulated data	Block design paradigm	Recursive least square estimation (RLSE) filtering	0.15~4.5 cm
Yamada et al., 2012	Primary motor	7	Finger tapping	Negative and positive correlation	1~4 cm
Frederick et al., 2012	Right frontal lobe	6	Resting state	Voxel-specific time delay	0.1 and 3 cm
Kamran and Hong, 2014	Motor	6	Finger tapping	ARMAX	~ 2.5 cm
Erdogan et al., 2014	Pre-frontal	18	Mental arithmetic	Extended superficial signal regression method.	2.5 cm
Kirilina et al., 2013	Frontal lobe	15	German words recognition	Time-domain fNIRS with wavelet coherence analysis.	3 cm
Bauernfeind et al., 2014	Motor cortex	12	Cue-based right hand (RH) and both feet (FE) motor execution	A common reference method, ICA and transfer function models	3 cm
Zhang et al., 2013	Five layer slab model	Simulated data	Epoch block	Multi-distance probe configuration and ICA	0.5 and 4.5 cm
Barker et al., 2013	Pre-frontal	22	Resting state	Regression analysis using GLM	–
Tong et al., 2013	Middle hand and left Big toe	7	Resting state	Group ICA	1.5 cm
Santosa et al., 2013	Pre-frontal	8	Arithmetic task	ICA with pre-defined regressors	2.2, 2.5, and 4.3 cm
Scarpa et al., 2013	Motor	10	Key pressing with left or right index finger	Reference channel based noise removal	1.5 and 3 cm
Kirilina et al., 2012	Pre-frontal	15	Semantic Continuous performance task	Concurrent fNIRS and fMRI with Bio-signals	3 cm
Cooper et al., 2012	Frontal and temporal lobe	7	Resting state	Variance of residues in GLM for concurrent fNIRS and fMRI	1 and~ 3cm
Hu et al., 2010	Motor	5	Finger tapping	Kalman filters and GLM	2 cm
Scarpa et al., 2010	Parieto-occipital	13	Visual graphics	Bayesian filtering	3 cm
Katura et al., 2008	Sensrimotor	30	Finger tapping	ICA	3 cm
Saager and Berger, 2008	Left pre-frontal	21	Resting state	Multi-detector CW-fNIRS	3.3 cm
Zhang et al., 2005	Sensrimotor	10	Finger movement task	Eigen vector based spatial filtering	> 3 cm
Cui et al., 2010	Motor	10	Finger tapping with head motion	Maximization of negative correlation	
Haeussinger et al., 2011	Frontal	24	Working memory	Identification of channels with major extra-cranial signal contributions	

### Resting state functional connectivity

Human brain generates continuous low frequency fluctuations during resting state. These low frequency fluctuations could be used as an informative source to understand the mechanism of different brain regions. The reason that these fluctuations are useful because they are correlated with different brain regions. These findings opened up a new way of thinking to explore a topic named as resting state functional connectivity (RSFC). There is no consensus available at the moment to specifically designating a range of frequencies for resting state low frequency fluctuations of hemodynamic measured waveform (Fox and Raichle, 2007; Lu et al., 2010). fMRI studies have reported a high level of inter-hemispheric correlations in different brain regions (Damoiseaux et al., 2006; De Luca et al., 2006). White et al. (2009) have reported their analysis regarding RSFC in motor in visual brain cortices. They calculated correlations using Pearson correlation coefficient. Their results suggest inter-hemispheric correlations exist in both motor and visual networks. Lu et al. (2010) analyzed the RSFC maps of the sensory motor and the auditory cortices using seed-based correlation analysis and data driven cluster analysis during resting state and motor-localizer task sessions. Their results suggested RSFCs were detected both within the ipsilateral and between the bilateral sensorimotor seed-regions. Additionally, it was found that significant correlation exist within the ipsilateral and between the bilateral temporal auditory cortices but not between temporal auditory areas. Zhang et al. (2011a) raised an issue of reliable RSFC maps. They analyzed test-reset reliability at three different scales; maps cluster and channel wise at individual and group levels. Their finding suggest that one should be very careful when interpreting the individual channel wise RSFC. But individual level and group level RSFC has excellent map-/cluster wise reliability. The trial-to-trial variability (TTV) in fNIRS signal exist even if the experimental procedure is kept constant. Hu et al. (2013) suggested to reduce TTV using RSFC information. They concluded that low frequency fluctuations are significant source of TTV and TTV decreases after removing the effects of bilateral connectivity. Since, fNIRS optodes cannot cover whole head surface which makes it difficult to analyze the RSFCs between all different brain regions (Lu et al., 2010). One possible way of covering whole skull is to increase the number of optodes. But this shall affect the temporal resolution of the equipment.

### Environmental and instrumental effects/artifacts/noises

fNIRS measured data series includes hemodynamic signal related to certain artifacts. These artifacts could be related to biological processes or from outer sources. Instrumental noise is one of major external sources whose affect could be reduced/nullify by proper calibration of the instrument. The other artifacts could be as a result of not good contact between skull and NIRS optodes. The uncoupling of optodes and skull is the source of fluctuations in the detected intensity, leading to uncorrected results. Thus, it must be insured that optodes and skull have a proper contact at correct angle. Removal of unnecessary hair at contact could also improve the quality of the signal. Another artifact is due the motion (tilt in the body, slight head moment, and breath holding) of the subject. This motion cause changes in the blood flow which is a major reason of fluctuations in the measured hemodynamic response. A crude way to remove motion artifacts is to average a certain number of experiments that its effects could be nullified. But for real-time BCI applications, it is necessary to estimate the actual contributions of the motion artifacts in single-trial. Yamada et al. (2009) presented a theoretical analysis of optical signal using Monte Carlo simulation. They proposed a multi-distant probe arrangement can reduce/eliminate artifacts in fNIRS measured data. Later, Robertson et al. (2010) experimented in a co-located channel configuration to analyze the known motion artifacts around three axis. They found motion related hemodynamic signal is detectable at co-located channels but not at unique channel. Cui et al. (2010) studied the effect of artifacts on fNIRS data induced by the head motion and they found that oxy- and deoxy-Hb are generally negatively correlated, head motion causes the correlation to become more positive. They proposed a correlation based signal improvement method to maximize the negative correlation between oxy- and deoxy-Hb signals. Haeussinger et al. (2011) developed a method to identify channels with major extra-cranial signal contributions and subtracted the average of these channels from all channels to obtain improved fNIRS signals. Thus, it would be concluded that moment in different direction caused changes in the absorption of light at different brain regions.

### Statistical significance and functional maps

fNIRS signal are highly corrupted by several measurement noises and physiological interferences. Therefore, a careful statistical analysis is required to extract neuronal-activity related signal from observed optical data (Tak and Ye, 2013; Kamran and Hong, 2014). The existence of a particular response *HRF*(*k*) in the measured data is found by a statistical analysis known as *t*-test (Hu et al., 2010; Kamran and Hong, 2013; Santosa et al., 2013, 2014; Hong and Nugyen, 2014). The basic idea is to test whether the estimated value of the activity strength parameter is greater or less than a target value zero with statistically significance (*t*-value > *t*_critical_ and *p* < 0.05). Thus, it is equivalent of testing a null hypothesis Ho with proper statistics i.e.,

Null hypothesis *H*_*o*_ : β_1_ = 0 (16)

Alternative hypothesis: β_1_ ≠ 0.

Finally, *t*-value could be evaluated as under

(16) $$\begin{array}{l} t_{value}=\frac{\beta_{1}-0}{SE\left(\beta_{1}\right)}. \end{array}$$

where SE is the standard error of the estimated coefficient.

However, in practical situations while analyzing fNIRS data, multiple comparison problems are often required to be addressed. Thus, there is chance that such analysis shall result in increase of inference error. Therefore, in addition to analyze the multi-data gathered through fNIRS modality, it is also required to put necessary checks that each individual data is analyzed with more care and strong level of evidence so that to reduce inference error in proceeding steps. For example, in Plichta et al. (2006), the Bonferroni correction and the Dubey/Armitage–Parmar alpha boundary were used for statistical inference of activated channels to estimate the statistical significance of fNIRS response during task periods. A detailed review on statistical analysis of fNIRS data could be found in Ye et al. (2009) and Tak and Ye (2013).

#### Challenges

fNIRS signal is not consistent among subjects, repeated trails and repetition of experiment even if the conditions are assumed to be similar. Therefore, the optical signal model constitutes an additional challenge to the researchers working in this area. Since, fNIRS time series is a combination of several physiological signals (Hu et al., 2010). These physiological signals are cardiac beat (~ 1 Hz), respiratory rhythm (~ 0.2–0.3 Hz) and low frequency fluctuations (< 0.1 Hz) among others. The first step is to estimate the frequency of particular signal present in the observed time series. In addition to this, it is also required to estimate the amplitudes of the signal present in the data. The next target is to find out the attributes of the HRF. There are several important characteristics, e.g., initial dip, FWHM, time to peak, peak height, post stimulus undershoot etc. Thus, a non-linear model is required to incorporate such attributes. For instant, two Gamma function model is most attractive because each Gamma function represents each peak (actual response and post dip). Furthermore, an iterative non-linear optimization algorithm is needed to estimate the free parameters in the model with significant accuracy. Finally, more precise statistical support is required to state that the estimation in the model is statistically significant. Another important factor is the design of experimental paradigm. The frequency in experimental paradigm must be different from physiological signals to avoid harmonics. Brain functionality is complex and coupled non-linear system. The analysis of different brain region's coupling is also a fundamental step toward improvement of fNIRS analysis. Considering the fact that fNIRS optodes cannot cover full skull, the number of optodes could be increased but it shall reduce the temporal resolution as well. Thus, an optimal source-detector separation is needed to establish for significant and maximum surface analysis.

## Conclusion

Brain engineering is a multi-disciplinary field with a focus to extract useful information from cortical signal observed by neuroimaging equipment. In this article recent advancements in the analysis of the optical signal observed through fNIRS are summarized. It is important for new researcher to understand the importance of pre-processing steps, effects of DPF and other factors during analysis. The recent conclusion for such factor is also presented. Additionally, different methodologies that have been developed in past to extract the neuronal activation related waveform (pre-processing steps, effects of DPF, variations and attributes of hemodynamic response function (HRF), extraction of evoked response, removal of physiological noises, instrumentation, and environmental noises, and resting/activation state functional connectivity), are summarized. Since systemic, instrumentation, and environmental noises effect the measured signal and the analysis. Therefore, reduction/removal of such noises must be performed carefully. Special consideration must be given for the selection of experimental paradigm to avoid physiological harmonics. Several methodologies that have been reported in past decade for noise removal, have also been summarized here. It is well-known by fMRI studies that different brain regions have connections during resting and task periods. Thus, it is very important to analyze such connections of brain using fNIRS as well. A brief review of RSFC is also added in this article.

## Author contributions

MK did the literature review and wrote the paper. MN participated in literature review and revising the article. MJ has suggested the theoretical aspects of the current study and supervised all the process from the beginning. All the authors have approved the final manuscript.

### Conflict of interest statement

The authors declare that the research was conducted in the absence of any commercial or financial relationships that could be construed as a potential conflict of interest.

## References

[B1] Abdelnour A. F. Huppert T. (2009). Real-time imaging of human brain function by near-infrared spectroscopy using an adaptive general linear model. Neuroimage 46, 133–143. 10.1016/j.neuroimage.2009.01.033 19457389 PMC2758631

[B2] Aqil M. Hong K.-S. Jeong M.-Y. Ge S. S. (2012a). Cortical brain imaging by adaptive filtering of NIRS signal. *Neurosci*. Lett. 514, 35–41. 10.1016/j.neulet.2012.02.048 22395086

[B3] Aqil M. Hong K.-S. Jeong M.-Y. Ge S. S. (2012b). Detection of event-related hemodynamic response to neuroactivation by dynamic modeling of brain activity. Neuroimage 63, 553–568. 10.1016/j.neuroimage.2012.07.006 22796989

[B4] Barati Z. Zakeri I. Pourrezaei K. (2013). Functional data analysis view of functional near infrared spectroscopy data. J. Biomed. Opt. 18:117007. 10.1117/1.jbo.18.11.117007 24247748

[B5] Barker J. W. Arabi A. Huppert T. J. (2013). Autoregressive model based algorithm for correcting motion and serially correlated errors in fNIRS. Biomed. Opt. Express 4, 1367–1379. 10.1364/BOE.4.001366 24009999 PMC3756568

[B6] Bauernfeind G. Wreissnegger S. C. Daly I. Müller-Putz R. (2014). Separating heart and brain: on the reduction of physiological noise from multichannel functional near-infrared spectroscopy. J. Neural Eng. 11:056010. 10.1088/1741-2560/11/5/056010 25111822

[B7] Boynton G. M. Engel S. A. Glover G. H. Heeger D. J. (1996). Linear systems analysis of functional magnetic resonance imaging in human V1. J. Neurosci. 16, 4207–4221. 8753882 10.1523/JNEUROSCI.16-13-04207.1996 PMC6579007

[B8] Chang P.-H. Lee S.-H. Gu G. M. Lee S.-H. Jin S.-H. Yeo S. S. (2014). The cortical activation pattern by are habilitation robotic hand: a functional NIRS study. Front. Hum. Neurosci. 8:49 10.3389/fnhum.2014.00049 PMC3915242 24570660

[B9] Ciftçi K. Sankur B. Kahya Y. P. Akin A. (2008). Multilevel statistical inference from functional near-infrared spectroscopy data during Stroop interference. IEEE Trans. Biomed. Eng. 55, 2212–2220. 10.1109/TBME.2008.923918 18713690

[B10] Cooper C. E. Elwell C. E. Meek J. H. Matcher S. J. Wyatt S. J. Cope M. (1996). The noninvasive measurement of absolute cerebral deoxy-hemoglobin concentration and mean optical path length in the neonatal brain by second derivative near infrared spectroscopy. Pediatr. Res. 39, 32–38. 10.1203/00006450-199601000-00005 8825383

[B11] Cooper R. J. Gagnon L. Goldenholz D. M. Boas D. A. Greve D. N. (2012). The utility of near-infrared spectroscopy in the regression of low-frequency physiological noise from functional magnetic resonance data. Neuroimage 59, 3128–3138. 10.1016/j.neuroimage.2011.11.028 22119653 PMC3288700

[B12] Cope M. Delpy D. T. (1988). System for long-term measurement of cerebral blood and tissue oxygenation on newborn-infants by near-infrared trans-illumination. Med. Biol. Eng. Comput. 26, 289–294. 10.1007/BF02447083 2855531

[B13] Cui X. Bray S. Reiss A. L. (2010). Functional near-infrared spectroscopy (NIRS) signal improvement based on negative correlation between oxygenated and deoxygenated hemoglobin dynamics. Neuroimage 49, 3039–3046. 10.1016/j.neuroimage.2009.11.050 19945536 PMC2818571

[B14] Damoiseaux J. S. Rombouts S. A. R. B. Barkhof F. Scheltens P. Stam C. J. Smith S. M.. (2006). Consistent resting-state networks across healthy subjects. Proc. Natl. Acad. Sci. U.S.A. 103, 13848–13853. 10.1073/pnas.0601417103 16945915 PMC1564249

[B15] De Luca M. Beckmann C. F. De Stefano N. Matthews P. M. Smith S. M. (2006). fMRI resting state networks define distinct modes of long-distance interactions in the human brain. Neuroimage 29, 1359–1367. 10.1016/j.neuroimage.2005.08.035 16260155

[B16] Duncan A. Meek J. H. Clemence M. Elwell C. E. Fallon P. Tyszczuk L.. (1996). Measurement of cranial optical path length as a function of age using phase resolved near infrared spectroscopy. Pediatr. Res. 39, 889–894. 10.1203/00006450-199605000-00025 8726247

[B17] Duncan A. Meek J. H. Clemence M. Elwell C. E. Tyszczuk L. Cope M. (1995). Optical path length measurements on adult heads, calf and forearms and the head of new born infants using phase resolved optical spectroscopy. Phys. Med. Biol. 40, 295–304. 10.1088/0031-9155/40/2/007 7708855

[B18] Erdogan S. B. Yucal M. A. Akin A. (2014). Analysis of task evoked systemic interferences in fNIRS measurements: insight from fMRI. Neuroimage 87, 490–504. 10.1016/j.neuroimage.2013.10.024 24148922

[B19] Fox M. D. Raichle M. E. (2007). Spontaneous fluctuations in brain activity observed with functional magnetic resonance imaging. Nat. Rev. Neurosci. 8, 700–711. 10.1038/nrn2201 17704812

[B20] Frederick B. de B. Nickerson L. D. Tong Y. (2012). Physiological de-noising of BOLD fMRI data using regressor interpolation at progressive time delays (RIPTiDe) processing of concurrent fMRI and near-infrared spectroscopy (NIRS). Neuroimage 60, 1913–1923. 10.1016/j.neuroimage.2012.01.140 22342801 PMC3593078

[B21] Friston K. J. Fletcher P. Joseph O. Holmes A. Rugg M. D. Tunner R. (1998). Event-related fMRI: characterizing differential responces Neuroimage 7, 30–40. 10.1006/nimg.1997.0306 9500830

[B22] Friston K. J. Holmes A. P. Worsley J.-P. Frith C. D. Frackowiak R. S. J. (1994). Statistical parameter maps in functional imaging: a general linear model approach. Hum. Brain Map. 2, 189–210. 10.1002/hbm.460020402

[B23] Gervain J. Mehler J. Werker J. F. Nelson C. A. Csibra G. Lloyd-Fox S.. (2011). Near-infrared spectroscopy: a report from the McDonnell infant methodology consortium. Dev. Cogn. Neurosci. 1, 22–46. 10.1016/j.dcn.2010.07.004 22436417 PMC6987576

[B24] Haeussinger F. B. Heinzel S. Hahn T. Schecklmann M. Ehlis A. C. Fallgatter A. J. (2011). Simulation of near-infrared light absorption considering individual head and prefrontal cortex anatomy: implications for optical neuroimaging. PLoS ONE 6:e26377. 10.1371/journal.pone.0026377 22039475 PMC3200329

[B25] Heinzel S. Haeussinger F. B. Hahn T. Ehlis A.-C. Plichta M. M. Fallgatter A. J. (2013). Variability of (functional) hemodynamics as measured with simultaneous fNIRS and fMRI during inter-temporal choice. Neuroimage 71, 125–134. 10.1016/j.neuroimage.2012.12.074 23313421

[B26] Herff C. Heger D. Fortmann O. Hennrich J. Putze F. Schultz T. (2014). Mental work load during n-back task-quantified in the prefrontal cortex using fNIRS. Front. Hum. Neurosci. 7:935 10.3389/fnhum.2013.00935 PMC3893598 24474913

[B27] Hong K.-S. Nugyen H.-D. (2014). State-space models of impulse hemodynamic responses over motor, somatosensory, and visual cortices. Biomed. Opt. Express 5, 1778–1798. 10.1364/BOE.5.001778 24940540 PMC4052911

[B28] Hu X. S. Hong K.-S. Ge S. S. (2012). fNIRS-based online deception decoding. J. Neural Eng. 9:026012. 10.1088/1741-2560/9/2/026012 22337819

[B29] Hu X. S. Hong K.-S. Ge S. S. (2013). Reduction of trial-to-trial variations in functional near-infrared spectroscopy signals by accounting for resting-state functional connectivity. J. Biomed. Opt. 18:117003 10.1117/1.JBO.18.1.017003 23291618

[B30] Hu X. S. Hong K.-S. Ge S. S. Jeong M. Y. (2010). Kalman estimator-and general linear model-based on-line brain activation mapping by near-infrared spectroscopy. BioMed. Eng. Online 9:82. 10.1186/1475-925x-9-82 21138595 PMC3020171

[B31] Jasdzewski G. Starngman G. Wanger J. Kwong K. K. Poldrack R. A. Boas D. A. (2003). Differences in the hemodynamic response to event-related motor and visual paradigms as measured by near-infrared spectroscopy. Neuroimage 20, 479–488. 10.1016/S1053-8119(03)00311-2 14527608

[B32] Jobsis F. F. (1977). Noninvasive, infrared monitoring of cerebral and myocardial oxygen sufficiency and circulatory parameters Science 198, 1264–1267. 10.1126/science.929199 929199

[B33] Kamran M. A. Hong K.-S. (2013). Linear parameter-varying model and adaptive filtering technique for detecting neuronal activities: an fNIRS study. J. Neural Eng. 10:056002. 10.1088/1741-2560/10/5/056002 23893789

[B34] Kamran M. A. Hong K.-S. (2014). Reduction of physiological effects in fNIRS waveforms for efficient brain-state decoding. Neurosci. Lett. 580, 130–136. 10.1016/j.neulet.2014.07.058 25111978

[B35] Kamran M. A. Jeong M.-J. Mannan M. M. N. (2015). Optimal Hemodynamic response model for functional near-infrared spectroscopy Front. Behav. Neurosci. 9:151. 10.3389/fnbeh.2015.00151 26136668 PMC4468613

[B36] Katura T. Sato H. Fuchino Y. Yoshida T. (2008). Extracting task-related activation components from optical tomography measurement using independent component analysis. J. Biomed. Opt. 13:054008 10.1117/1.2981829 19021388

[B37] Khan M. J. Hong M. J. Hong K.-S. (2014). Decoding of four movement directions using hybrid NIRS-EEG brain-computer interface. Front. Hum. Neurosci. 8:244. 10.3389/fnhum.2014.00244 24808844 PMC4009438

[B38] Kirilina E. Jelzow A. Heine A. Niessing M. Wabnitz H. Brühl R.. (2012). The physiological origin of task-evoked systemic artefacts in functional near infrared spectroscopy. Neuroimage 61, 70–81. 10.1016/j.neuroimage.2012.02.074 22426347 PMC3348501

[B39] Kirilina E. Yu N. Jelzow A. Wabnitz H. Jacobs A. M. Tachtsidis I. (2013). Identifying and quantifying main components of physiological noise in functional near infrared spectroscopy on the prefrontal cortex. Front. Hum. Neurosci. 7:864. 10.3389/fnhum.2013.00864 24399947 PMC3865602

[B40] Koh P. H. Glaser D. E. Flandin G. Butterworth B. Maki A. Delpy D. (2007). Functional Optical Signal Analysis (fOSA): a software tool for NIRS data processing incorporating Statistical Parametric Mapping (SPM). J. Biomed. Opt. 12:064010 10.1117/1.2804092 18163826

[B41] Kohl M. Nolte C. Heekeren H. R. Horst S. Scholz U. Obrig H.. (1998). Determination of the wavelength dependence of the differential pathlength factor from near-infrared pulse signals. Phys. Med. Biol. 43, 1771–1782. 10.1088/0031-9155/43/6/028 9651039

[B42] Kopton I. M. Kenning P. (2014). Near-infrared spectroscopy as new tool for neuroscience research. Front. Hum. Neurosci. 8:549 10.3389/fnhum.2014.00549 25147517 PMC4124877

[B43] Koray C. Bulent S. Yasemin P. K. Ata A. (2008). Constraining the general linear model for sensible hemodynamic response function waveforms. Med. Biol. Eng. Comput. 46, 779–787. 10.1007/s11517-008-0347-6 18427851

[B44] Lu C.-M. Zhang Y.-J. Biswal B. B. Zang Y.-F. Peng D.-L. Zhu C.-Z. (2010). Use of fNIRS to assess resting state functional connectivity. J. Neurosci. Methods 186, 242–249. 10.1016/j.jneumeth.2009.11.010 19931310

[B45] Machado A. Lina J. M. Tremblay J. Lassonde M. Nguyen D. K. Lesage F. (2011). Detection of hemodynamic responses to epileptic activity using simultaneous Electro-EncephaloGraphy (EEG)/Near Infrared Spectroscopy (NIRS) acquisitions. Neuroimage 56, 114–125. 10.1016/j.neuroimage.2010.12.026 21168514

[B46] Molavi B. May L. Gervain J. Carreiras M. Werker J. F. Dumont G. A. (2014). Analysing the resting state functional connectivity in the human language system using near infrared spectroscopy. Front. Hum. Neurosci. 7:921 10.3389/fnhum.2013.00921 24523685 PMC3905209

[B47] Morren G. Wolf U. Lemmerling P. Wolf M. Choi J. H. Gratton E.. (2004). Detection of fast neuronal signals in the motor cortex from functional near infrared spectroscopy measurements using independent component analysis. Med. Biol. Eng. Comput. 42, 92–99. 10.1007/BF02351016 14977228

[B48] Naseer N. Hong K.-S. (2015). fNIRS-based brain-computer interfaces: a review. Front. Hum. Neurosci. 9:3. 10.3389/fnhum.2015.00003 25674060 PMC4309034

[B49] Plichta M. M. Heinzel S. Ehlis A. C. Pauli P. Fallgatter A. J. (2007). Model-based analysis of rapid event-related functional near-infrared spectroscopy (NIRS) data: a parametric validation study. Neuroimage 35, 625–634. 10.1016/j.neuroimage.2006.11.028 17258472

[B50] Plichta M. M. Herrmann M. J. Ehlis A. C. Baehne C. G. Richter M. M. Fallgatter A. J. (2006). Event-related visual versus blocked motor task: detection of specific cortical activation patterns with functional near-infrared spectroscopy. Neuropsychobiology 53, 77–82. 10.1159/000091723 16511338

[B51] Power S. D. Kushki A. Chau T. (2012). Intersession consistency of single-trial classification of the prefrontal response to mental arithmetic and the non-control state by NIRS. PLoS ONE 7:e37791 10.1371/journal.pone.0037791 22844390 PMC3402505

[B52] Prince S. J. D. Kolehmainen V. Kaipio J. P. Franceschini M. A. Boas D. Arridge S. R. (2003). Time series estimation of biological factors in optical diffusion tomography. Phys. Med. Biol. 48, 1491–1504. 10.1088/0031-9155/48/11/301 12817933

[B53] Robertson F. C. Douglas T. S. Meintjes E. M. (2010). Motion artifact removal for functional near-infrared spectroscopy: a comparison of methods. IEEE Trans. Biomed. Eng. 57, 1377–1387. 10.1109/TBME.2009.2038667 20172809

[B54] Saager R. Berger K. (2008). Measurment of layer-like hemmodynamic trend in scalp and cotcex: implication for phsiological baseline suppression in functional near-infrared spectroscopy. J. Biomed. Opt. 13:034017 10.1117/1.2940587 18601562

[B55] Santosa H. Hong M. J. Hong K.-S. (2014). Lateralization of music processing with noises in the auditory cortex: an fNIRS study. Front. Behav. Neurosci. 8:418. 10.3389/fnbeh.2014.00418 25538583 PMC4260509

[B56] Santosa H. Hong M. J. Kim S.-P. Hong K.-S. (2013). Noise reduction in functional near-infrared spectroscopy signals by independent component analysis. Rev. Sci. Instrum. 84:073106. 10.1063/1.4812785 23902043

[B57] Scarpa F. Brigadoi S. Cutini S. Scatturin P. Zorzi M. Dell'acqua R.. (2013). A reference-channel based methodology to improve estimation of event-related hemodynamic response from fNIRS measurements. Neuroimage 72, 106–119. 10.1016/j.neuroimage.2013.01.021 23357074

[B58] Scarpa F. Cuitni S. Scatturin P. Dell'Aqua R. Sparacino G. (2010). Bayesian filtering of human brain hemodynamic activity elicited by visual short-term maintenance recorded through functional near-infrared spectroscopy (fNIRS) Opt. Express 18, 26550–26568. 10.1364/OE.18.026550 21165006

[B59] Scholkmann F. Wolf M. (2013). General equation for the differential pathlength factor of the frontal human head depending on wavelength and age. J. Bio. Opt. 18:105004. 10.1117/1.JBO.18.10.105004 24121731

[B60] Schroeter M. L. Zysset S. Kruggel F. von Cramon D. Y. (2003). Age dependency of the hemodynamic response as measured by functional near-infrared spectroscopy. Neuroimage 19, 555–564. 10.1016/S1053-8119(03)00155-1 12880787

[B61] Sitaram R. Caria A. Birbaumer N. (2009). Hemodynamic brain-computer interfaces for communication and rehabilitation. Neural Netw. 22, 1320–1328. 10.1016/j.neunet.2009.05.009 19524399

[B62] Taga G. Homae F. Watanab H. (2007). Effects of source-detector distance of near infrared spectroscopy on the measurement of the cortical hemodynamic response in infants. NeuroImage 7, 452–460. 10.1016/j.neuroimage.2007.07.050 17884584

[B63] Tak S. Ye J. C. (2013). Statistical analysis of fNIRS data: a comprehensive review. Neuroimage 85, 72–91. 10.1016/j.neuroimage.2013.06.016 23774396

[B64] Tong Y. Hocke L. M. Nickerson L. D. Licata S. C. Lindsey K. P. Frederick B. (2013). Evaluating the effects of systemic low frequency oscillations measured in the periphery on the independent component analysis results of resting state networks. Neuroimage 76, 202–215. 10.1016/j.neuroimage.2013.03.019 23523805 PMC3652630

[B65] Umeyama S. Yamada T. (2013). Detection of an unstable and/or a weak probe contact in a multichannel near-infrared spectroscopy measurement. J. Biomed. Opt. 18:047003. 10.1117/1.jbo.18.4.047003 23552638

[B66] van der Zee P. Cope M. Arridge S. R. Essenpreis M. Potter L. A. Edwards A. D. (1992). Experimentally measured optical path lengths for the adult head, calf and forearm and the head of the newborn infant as a function of inter optode spacing. Adv. Exp. Med. Biol. 316, 143–153. 10.1007/978-1-4615-3404-4_17 1288074

[B67] White B. R. Snyder A. Z. Cohen A. L. Petersen S. E. Raichle M. E. Schlaggar B. L.. (2009). Resting-state functional connectivity in the human brain revealed with diffuse optical tomography. Neuroimage 47, 148–156. 10.1016/j.neuroimage.2009.03.058 19344773 PMC2699418

[B68] Woolrich M. W. Behrens T. E. J. Smith S. M. (2004). Constrained linear basis sets for HRF modelling using variational Bayes. Neuroimage 21, 1748–1761. 10.1016/j.neuroimage.2003.12.024 15050595

[B69] Yamada T. Umeyama S. Matsuda K. (2009). Multidistance probe arrangement to eliminate artifacts in functional near-infrared spectroscopy. J. Biomed. Opt. 14:064034. 10.1117/1.3275469 20059272

[B70] Yamada T. Umeyama S. Matsuda K. (2012). Separation of fNIRS signals into functional and systemic components based on differences in hemodynamic modalities. PLoS ONE 7:e50271. 10.1371/journal.pone.0050271 23185590 PMC3501470

[B71] Ye J. C. Tak S. Jang K. E. Jung J. Jang J. (2009). NIRS-SPM: statistical parametric mapping for near-infrared spectroscopy. Neuroimage 44, 428–447. 10.1016/j.neuroimage.2008.08.036 18848897

[B72] Yunjie T. Blaise F. (2012). Concurrent fNIRS and fMRI processing allows independent visualization of the propagation of pressure waves and bulk blood flow in the cerebral vasculature. Neuroimage 61, 1419–1427. 10.1016/j.neuroimage.2012.03.009 22440649 PMC3376221

[B73] Zhang H. Zhang Y. J. Duan L. Ma S. Y. Lu C. M. Zhu C. Z. (2011a). Is resting-state functional connectivity revealed by functional near-infrared spectroscopy test-retest reliable? J. Biomed. Opt. 16:067008. 10.1117/1.3591020 21721829

[B74] Zhang Q. Brown E. N. Strangman G. E. (2007). Adaptive filtering for global interference cancellation and real-time recovery of evoked brain activity: a Monte Carlo simulation study. J. Bio. Opt. 12:044014. 10.1117/1.2754714 17867818

[B75] Zhang Y. Brooks D. H. Francischini N. A. Boas D. A. (2005). Eigen vector based spatial filtering for reduction of physiological interference in diffuse optical imaging. J. Biomed. Opt. 10:011014 10.1117/1.1852552 15847580

[B76] Zhang Y. Liu X. Yang C. Wang K. Sun J. Rolfe P. (2013). A new approach to separate hemodynamic signals for barin-computer interface using independent component analysis and least squares. J. Spectrosc. 2013:950302 10.1155/2013/950302

[B77] Zhang Y. Sun J. Rolfe P. (2011b). Reduction of global interference in functional multi-distance near-infrared spectroscopy using empirical mode decomposition and recursive least squares: a Monte Carlo study. J. Eur. Opt. Soc. 16:067008 10.2971/jeos.2011.11033

[B78] Zhang Y. Sun J. Rolfe P. (2012). RLS adaptive filtering for physiological interference reduction in NIRS brain activity measurement: a Monte Carlo study. Physiol. Meas. 33, 925–942. 10.1088/0967-3334/33/6/925 22551687

